# Molecular Biology in Treatment Decision Processes—Neuro-Oncology Edition

**DOI:** 10.3390/ijms222413278

**Published:** 2021-12-10

**Authors:** Andra V. Krauze, Kevin Camphausen

**Affiliations:** Radiation Oncology Branch, Center for Cancer Research, National Cancer Institute, NIH, 9000 Rockville Pike, Building 10, Bethesda, MD 20892, USA; camphauk@mail.nih.gov

**Keywords:** radiation oncology, neuro-oncology, computational, machine learning, deep learning and artificial intelligence, molecular biomarkers

## Abstract

Computational approaches including machine learning, deep learning, and artificial intelligence are growing in importance in all medical specialties as large data repositories are increasingly being optimised. Radiation oncology as a discipline is at the forefront of large-scale data acquisition and well positioned towards both the production and analysis of large-scale oncologic data with the potential for clinically driven endpoints and advancement of patient outcomes. Neuro-oncology is comprised of malignancies that often carry poor prognosis and significant neurological sequelae. The analysis of radiation therapy mediated treatment and the potential for computationally mediated analyses may lead to more precise therapy by employing large scale data. We analysed the state of the literature pertaining to large scale data, computational analysis, and the advancement of molecular biomarkers in neuro-oncology with emphasis on radiation oncology. We aimed to connect existing and evolving approaches to realistic avenues for clinical implementation focusing on low grade gliomas (LGG), high grade gliomas (HGG), management of the elderly patient with HGG, rare central nervous system tumors, craniospinal irradiation, and re-irradiation to examine how computational analysis and molecular science may synergistically drive advances in personalised radiation therapy (RT) and optimise patient outcomes.

## 1. Introduction

Computational approaches including machine learning, deep learning, and artificial intelligence are growing in importance in all medical specialties as large data repositories are increasingly being curated and optimised. Radiation Oncology is a specialty that has evolved to deliver highly precise and accurate treatment to tumors surrounded by normal tissue and is at the forefront of large-scale data acquisition in Oncology. As such, Radiation Oncology is well positioned for both the production and analysis of large-scale oncologic data with the potential for clinically driven endpoints and advancement of patient outcomes. Neuro-oncology is comprised of malignancies that often carry poor prognoses and significant neurological sequelae as well as the potential for life altering acute and late effects [1]. These are also tumors that tend to recur in the radiation field emphasizing the need to understand tumor radiosensitivity and resistance to treatment both of which cannot be robustly addressed by creating robust connections to molecular science. At the intersection of Radiation Oncology and neuro-oncology, a number of literature reviews [2,3,4], and original studies [5,6,7] have explored computationally mediated research that may eventually lead to precision management by employing large scale datasets. This review analysed the state of the literature pertaining to large scale data and computational analysis in neuro-oncology with emphasis on Radiation Oncology and connected the existing and evolving approaches in molecular biomarker development to realistic avenues for clinical implementation. In particular, the focus was on specific clinical areas of controversy: management of low-grade gliomas (LGG), high grade gliomas (HGG), the specific scenario of the elderly patient with HGG as well as rare central nervous system tumors, craniospinal irradiation, and re-irradiation with a proposal of how computational analysis may drive personalised radiation therapy (RT) to optimise patient outcomes and merge traditional radiation planning concepts with improved molecular profiling.

## 2. Computational Analysis in Radiation Therapy Treatment Planning—Current State

**Figure 1:**
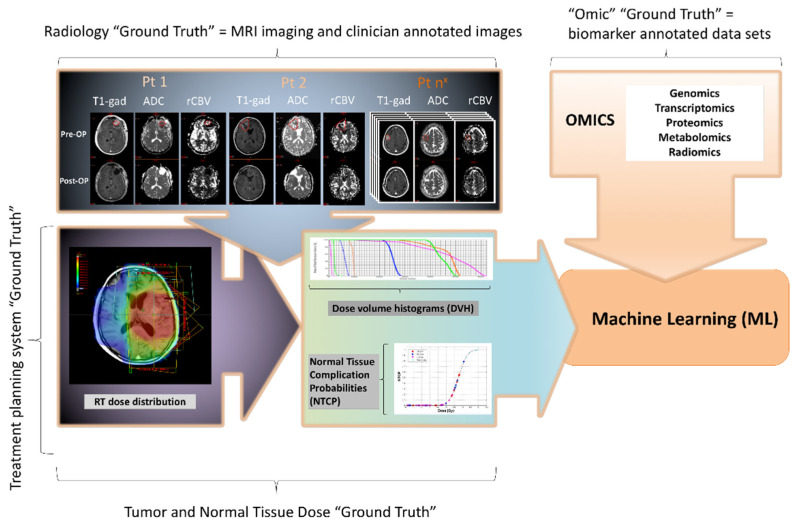
Harnessing large-scale neuro-oncology data and computational analysis to achieve precision neuro-oncology in RT. Top left panel: Tumor volumes (as well as normal tissue) are delineated (contoured) on standard of care acquired MRIs of the brain which are co-registered with CT. The images are hence clinician annotated representing a form of ground truth that can be employed in training AI methods. Bottom left panel: the co-registration of MRI and CT allows for radiation therapy dose to be calculated to the structures that have been delineated allowing for ML approaches that can examine the relationship between tumor and normal tissues volumes, radiation therapy dose, tumor response and failure. Top right panel: Omics data of all subtypes can be aggregated with imaging and radiation therapy data to identify clinically meaningful biomarkers.

**Figure 2:**
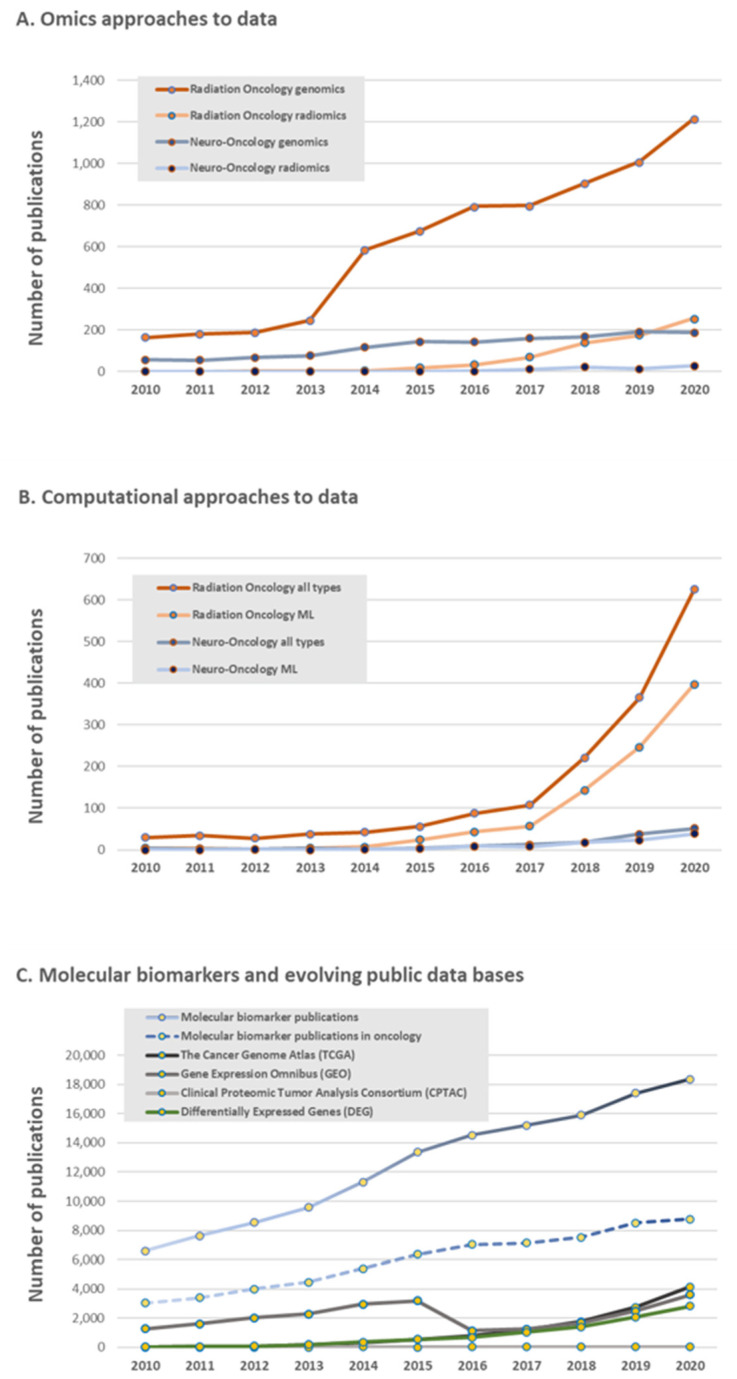
Cumulative number of related articles/studies in PUBmed 2010 to present (30 September 2021) aimed at (**A**) omics. (**B**) computational approaches and (**C**) molecular biomarkers and evolving public data bases grouped by Radiation oncology and Neuro-oncology as a discipline. Machine Learning (ML) approaches represent the most mature and most rapidly growing subset of computational analysis approaches overall. C. Molecular biomarkers and evolving public omic data sets resulting in analyses of differentially expressed genes (DEG) [36].

Radiation therapy deals with the administration of therapeutic radiation for mostly malignant and some nonmalignant conditions [8]. Radiation is aimed at specific targets or volumes delineated by the clinician using information originating in the patient history, physical exam and radiologic imaging. Data collected is represented by direct data entry fields (manual or automatic) in the treatment planning system (e.g., tumor site, treatment intent, ready to treat dates, patient setup and desired technique employed in treatment planning), as well as tumor and normal tissue volumes delineated by the clinician and RT dose delivered using multiple DVH (Dose Volume Histogram) (Figure 1). These large-scale datasets can be employed for administrative purposes, such as capturing the number of patients on treatment that share a common histology or planning technique, but are also most relevant to computational approaches that involve artificial intelligence (AI) [3,4,9,10,11,12,13], machine learning (ML) [2,4,5,6,7,9,13,14,15,16,17,18,19,20,21,22], deep learning (DL) [2,3,11,23,24,25,26,27], ground truth [7,13] and radiogenomics [2,13,14,28,29,30,31,32,33,34] (See Table 1 for definitions). Radiogenomics can allow for the relationship between dose delivered to tumor volumes and normal tissues and their response to be linked to clinician observed toxicity and tumor response as well as patient reported outcomes. Although the latter two parameters may be collected, it is often in a system separate from the treatment planning system itself and, thus, there is no direct communication between the two that would enable a seamless analysis. Noteworthy is the fact that whilst advancements have been made in terms of targeted systemic management, radiation therapy (RT) treatment volumes, doses, and fractionation for central nervous system (CNS) tumors have remained largely unaltered over time despite ongoing advances in molecular tumor characterization [35]. The parallel progress in radiomics and genomics has generated significant interest in the radiation oncology literature but less so in the context of neuro-oncology (Figure 2) [36]. Treatment planning in RT is based on generating volumes or targets using contrast enhanced MRI co-registered with CT simulation, with treatment volumes comprised of areas the radiation oncologist deems consistent with tumor presence on MRI (GTV or Gross tumor volume), adding margins for what may represent areas at risk of tumor involvement (CTV or Clinical Target Volume) and a geometric margin for setup variability and motion (PTV or Planning Target Volume) [8]. Relevant available imaging including metabolic imaging when available may also be employed to help generate the G, but no radiomic, genomic or radiogenomic approaches in either the general oncology context or the radiation oncology specific context (Table 1) are currently incorporated as standard of care [37]. The CTV in high grade glioma is comprised of the GTV on T1 gadolinium enhanced scans with a 2 cm geometric expansion in an attempt to capture what historically is suspected to represent subclinical disease. The PTV is subject to patient immobilisation and technique and is generally 5 mm on most linear accelerators and can be reduced in the context of stereotactic radiation therapy. In low grade glioma, a secondary CTV may be treated to a lesser dose if generated using the T2 FLAIR abnormality with a 1.5 cm margin. In some histologies with overt or significant risk for craniospinal dissemination, craniospinal irradiation may also be administered with additional dose or boost to areas of disease in the spine. The dose administered to high grade glioma is generally 60 Gy [38] and low-grade glioma 54 Gy [39] at 1.8–2.0 Gy/fr. Ongoing advancement in radiogenomics has not altered volumes, dose or fractionation in most CNS tumors. While commonly employed RT planning software such as Eclipse inherently collect both treatment volume information (clinician annotated data by contouring or outlining tumors based on patient imaging), as well as dose distribution (dose delivered to both the tumor targets and organs at risk in the field), the ability to incorporate treatment planning and dosimetry information into radiomics using common radiology platforms (e.g., PACS) is complex, lacking in the clinic and in limited use as yet in radiomic research [40]. This is due to barriers that include both the mining and the farming aspect of the data [40]. Key data elements may be missing and/or retrieval may be difficult ultimately requiring significant process changes and data transformation. Most computational studies aimed at radiation therapy volumes have instead focused on autogenerating volumes while accounting for neuroanatomy [41,42,43] but less so on molecular classification or the incorporation of radiogenomic techniques [4,6,23]. The link between dose distribution and dose to tumor volumes and organs at risk and normal tissue toxicity as well as radioresistance and radiosensitivity has only received limited exploration in the big data context at this time although active efforts are ongoing to data farm in anticipation of progress in this domain [44].

## 3. Low Grade Gliomas

**Figure 3:**
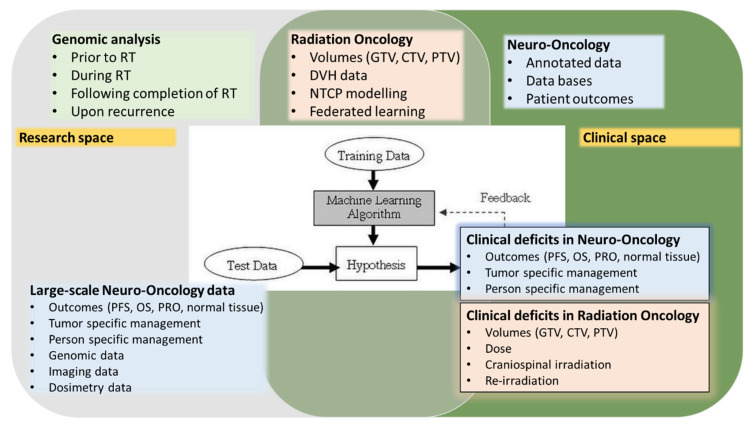
Workflow for large-scale neuro-oncology data and computational analysis towards the identification of clinically applicable biomarkers and precision neuro-oncology [55].

Diffuse low-grade gliomas (DLGGs, WHO Grade II gliomas), comprise 13–16% of all primary brain tumors [1]. The 2016 WHO classification of central nervous system tumors, unlike its predecessor, included molecular parameters as well as morphology to establish diagnosis [1]. Low grade gliomas (LGG) carry generally a superior prognosis as compared to high grade gliomas (HGG) and are defined both in terms of histology as well as their molecular classification. The diagnosis of oligodendroglioma and anaplastic oligodendroglioma requires demonstration of both an IDH gene family mutation and combined whole-arm losses of 1p and 19q (1p/19q codeletion) [1]. The management of patients with LGG usually involves resection followed by observation, radiation and or chemotherapy but remains heterogenous and is best carried out in a multidisciplinary setting [39,45,46,47]. The acute need to provide precision management for these tumors is enforced by the longevity that patients with LGG exhibit [47] and the need to minimise both acute and late toxicity secondary to chemotherapy and RT. To improve outcomes and personalise management, the neuro-oncology field has accurately recognised that both the need for tumor sequencing and computational interpretation of imaging are crucial [14]. However, efforts here have focused largely on diagnosis, grading and the type and timing of systemic management [28,48]. Molecular profiling has been evaluated in the CATNON and CODEL trials [49,50] to identify the appropriate chemotherapy to administer in conjunction with RT but has not been used prospectively to evaluate the ideal delivery of RT. LGG is a particularly challenging entity in that randomised trials may take many years to produce results [45,46], traditional endpoints such as overall survival and progression free survival may not reflect challenges in patient outcomes and hence observational large scale data may carry a large role if appropriately employed [48]. Research efforts in LGG have focused on two major areas: radiomics and genomics [3,6,14,28]. Radiomics aims to harness imaging features that would normally be subject to human interpretation to help classify gliomas [28]. In parallel genomic analysis of tumor samples provides a molecular profile [51]. Radiogenomics, at the intersection of these two approaches allows statistical correlations of radiomic features with genetic aberrations obtained from mutational analyses or next-generation sequencing data and has the potential to provide “virtual biopsy” maps [3] and the creation of radiogenomics pipelines can help define tumor biology [2]. Machine learning (ML) (Table 1) techniques, including deep learning, can thus be employed to identify a pattern that can predict and or refine tumor classification from MRI or MRI/PET [51,52,53,54] such as using MRI and deep learning to define 1p19q co-deletion in gliomas [52]. Existing approaches do suffer from lack of standardisation with respect to image acquisition, processing, segmentation, feature extraction, machine learning algorithm and validation and large ground truth data sets (Table 1 and Figure 3) [3,7,15,24,55]. Efforts towards standardisation are being made [56] but will require ongoing adjustment and mandated inclusion into clinical trial design. While some studies have examined radiomics in the context of treatment response [57], most available studies have largely focused on prognostic diagnosis, classification, and prognostic features [2,3,9]. As a result, current findings have yet to impact RT volumes, dose or fractionation [58] and the inclusion and standardisation of radiation related data has yet to be defined or implemented [4]. Late effects including data defining normal tissue toxicity, including impact on organs at risk in the RT field, and the development of neurocognitive changes in response to RT dose distribution, have undergone limited study [59,60,61]. Future trials and data farming in large scale registries would have to mandate a merger with RT data including volumes, dose, dose per fraction and dose distributions, to examine both patterns of failure and correlation with recurrent disease in conjunction with genomic and radiomic analyses as well as late effects [40]. This will allow for analysis of tumor response and a personalised approach to RT in LGG.

## 4. High Grade Gliomas

The majority of gliomas are high-grade (WHO grade III and IV), with the most common and aggressive form of glioma being Glioblastoma (GBM). GBM (WHO grade IV) represents over half (56.1%) of all gliomas [1]. In high grade gliomas the prognosis remains poor with 6% 5-year survival [1]. These tumors exhibit both significant heterogeneity and radioresistance [35,62]. HGG is managed with maximal safe resection followed by concurrent chemoirradiation [38]. Precision neuro-oncology can help personalise management to prolong life by gaining a deeper understanding of the location, extent and molecular classification and possible avenues of response of radioresistant disease. Approaches have involved studies aimed at methylation and its connection to imaging changes [16,17,25,63,64,65]. However, existing approaches are hampered by differences between primary and recurring tumors as well as links between DNA methylation, the tumor microenvironment, and the association of epigenetic tumor heterogeneity with patient survival in this genetically diverse and heterogenous malignancy [63]. An important avenue for research has centred on computational approaches with the challenge of distinguishing progression from pseudoprogression. Currently this distinction is subject to the RANO guidelines [66] but as radiomics, genomics and machine learning converge, it would be imperative to advance the existing RANO framework by including machine learning driven criteria for progression vs. pseudoprogression which are currently subject to clinical and radiographic interpretation. Recent publications [67], Kim [68], Ismail [69], Kebir [70], Akbari [71] focused on specific aspects, including only MRI imaging [68], only PET [70] or only histopathology [71] but few included the RT treatment fields [67,72]. Each of these approaches employed single institution data and small patient samples and hence reproducibility may prove challenging. Unfortunately, most proposed workflows that include radiogenomics in glioma stop short of including radiation therapy management or dosimetry [2,9,26,72]. In addition, significant challenges remain to safe data sharing and amalgamation of multiple sources of information that would allow for superior results from machine learning approaches [18].

## 5. Management of the Older Patient with High Grade Glioma—An Area of Controversy in the Clinic

Within the high-grade glioma patient cohort, the management of the elderly patient with glioblastoma remains particularly controversial [73,74] given that prognosis is particularly poor and the goal of care if often highly palliative in most patients. This is further complicated by poor representation in clinical trials [75]. In this patient cohort, defining the most appropriate level of aggressiveness of management to achieve the best outcome for the patient while balancing neurologic function, quality of life and longevity can at this point only be addressed robustly with real world data [76]. Real world data has been analysed concluding the importance of capturing performance status in order to facilitate analysis while attempts at generating prognostic scores have been made [76,77]. Poor capture of performance status in large scale data sets and more limited genomic analysis in the context of a higher biopsy rate versus resection rate, have hampered efforts to harness radiogenomic approaches in elderly patients with glioma. As of yet, radiogenomic approaches have not impacted precision management in the clinic in the elderly patient with high grade glioma.

## 6. Radiogenomic Advances in Rare CNS Histologies, Craniospinal, and Re-Irradiation Settings

Radiomics and radiogenomic driven approaches to radiation therapy in rare central nervous system tumors such as ependymoma, medulloblastoma, craniopharyngioma, Diffuse intrinsic pontine glioma (DIPG) are understandably even less advanced as compared to glioma and no attempts have been made to alter standard of care RT by leveraging large scale data, radiogenomics or computational approaches. Due to the rare nature of these tumors, large-scale real-world data is mostly lacking, and randomised data are almost impossible to acquire making computational approaches especially relevant. Most studies have focused on diagnosis in the context of CNS tumors where tissue acquisition is challenging or impossible such as pediatric posterior fossa tumors [19], rare histologies or histologies that present difficult diagnostic interpretation (ependymoma, pilocytic astrocytoma, medulloblastoma, craniopharyngioma) [10,20,27,78,79] and very few studies examined computational avenues to optimise RT [80], Zhu [81]. In meningioma attempts have focused on diagnosis and grading especially in the context of radiomics and surgical resection [21] and linked analysis to extent of tumor and brain or bone invasion [82,83,84,85]. If analysed in conjunction with biomarkers and RT dosimetry, these endeavors could prove highly relevant to RT volumes and dose as well patterns of recurrence [86,87,88] but clinical applications are only starting to emerge [89]. Attempts to alter RT volumes in future trials should focus on optimizing RT treatment planning in a manner consistent with radiogenomic driven pattern of failure analysis [90]. Other areas of ongoing debate include the administration of craniospinal irradiation and RT volumes in this scenario particularly in rare tumors with superior long-term prognosis where risk to normal tissue and late effects are particularly relevant. This is often a point of discussion in ependymoma and medulloblastoma but also in other rare histologies. No computational analysis has been identified in this scenario yet.

## 7. The Future of Molecular Science—Using AI and Big Data to Bring Molecular Biomarkers into the Clinic

We have observed significant progress in radiomics, genomics with significant growth in molecular biomarkers (Figure 2) [36]. Although clinically actionable information based on FDA approved biomarkers was most frequently observed in oncology, within oncology most of the research has focused on biomarker-drug pairs associated with cancer drugs compared to those for other therapeutic areas (*n* = 92 (59.7%) vs. *n* = 62 (40.3%), *p* < 0.0051) [91]. In histopathology, significant advances are ongoing [11] to extract biomarkers directly from histology images and summarise studies of basic and advanced image analysis for cancer histology. This includes inference of molecular features, prediction of survival and end-to-end prediction of therapy response. There are currently no FDA approved genomic markers related to RT. No relationships have yet been investigated with a biomarker lens with respect to RT dose, dose/fraction, volume of tumor and normal tissue. Radiogenomics aimed at oxic and hypoxic response gene signatures in conjunction with imaging and dosimetry [29] could help advance personalisation of RT volumes and dose to optimise outcomes. Re-irradiation upon tumor recurrence represents another instance where data aimed at balancing response and toxicity is lacking [30,31,32,33]. While technological advances have improved the ability to target tumor recurrence while limiting dose to organs at risk, radiosensitivity of both malignant and normal tissues remain subject of ongoing investigations. In the clinic, dose constraints currently employed are based on large dated series with significant limitations with respect to ability to compare dose per fraction and limited robustly collected normal tissue complication probability data particularly in the CNS and in the context of SRS and SRT [59,60,61,92]. There is significant potential in examination of tumor as well as normal tissue response with respect to gene expression profiling, lymphocyte assays, radiogenomics with structural variations including single nucleotide polymorphisms (SNPs), copy number variations (CNVs), gene expression (mRNA, miRNA, lncRNA) [33,34]. In this context the challenge of leveraging existing molecular data that is increasingly curated in large scale public data bases (Figure 2C) including The Cancer Genome Atlas Program (TCGA) [93,94,95], Gene Expression Omnibus (GEO) [96], the growing presence of the Clinical Proteomic Tumor Analysis Consortium (CPTAC) [97] lies in both merging the acquisition of further data towards robust clinical endpoints as well as merging dose volume histogram and pattern of failure data present currently in distinct silos with exisiting results [29,98,99,100,101]. These efforts are gaining ground at the trascriptome [99,100,102] and proteome [97] levels with paralleled progress in the identification of differentially expressed genes (DEG) (Figure 2C). However, further advancement will require robust frameworks and workflows in the clinical space that allow for continual linking to the research space (Figure 3) [55]. Future trials would do well to also consistently incorporate functional and novel imaging that define both tumor response and normal tissue toxicity and link acquired data to evolving molecular biomarkers in order to succeed in generating a radiogenomic-based personalised approach, particularly as data becomes more abundant.

This would be needed in conjunction with robust “at the elbow” connection of all relevant data (pathology, imaging, genomic analysis and RT dosimetry of both tumor and normal tissue) [44]. This may be advanced by endorsing the pooling of data across large collaborative teams and embracing the diversity of the data that is likely to come forth [34]. Attempts at this are ongoing with the Radiogenomics Consortium (RGC) [34], ReSPOND consortium [12], RadiationGeneSigDB [29], MarkerDB [103], and federated learning [104]. Clinicians will need to exercise significant ownership outlining transferable, lasting, clinically driven endpoints to be captured including a pluralistic definition of progression and response that also includes the impact on normal tissues and neurocognitive function.

## 8. Conclusions

The current nuances involved in the molecular classification of central nervous system tumors are not reflected in the current volumes treated in radiation therapy due to lack of biomarkers supporting a change in standard of care volumes and fractionation and a lack of robust understanding of differential radiosensitivity or resistance. Advances in radiogenomics may well provide the needed evidence to allow for more personalised dose distributions, which are highly achievable with current technology. Increased emphasis needs to be placed on biologically optimising RT to improve outcomes with the solid backing of molecular science. Current prospective protocols call for standard for care radiation therapy even as they advance the acquisition of tumor sequencing and radiogenomics by attempting to standardise both imaging and mandate tumor sequencing. Yet robust links between the dose volume distribution generated by the RT treatment plan are often divorced from genomic and radiomics analysis. Growing large scale repositiories at all omics levels will make such link possible. Precision neuro-oncology as it relates to radiation oncology lies currently in the more distant future due to limited data sharing and lack of robust implementation of radioresistance and radiosensitivity as we follow ongoing growth of molecular biomarkers. These carry unsurpassed potential for clinically meaningful outcomes and will need to be incorporated as end points into clinical trials and large-scale registries and prioritised in clinical trial design to allow for true advancement in this space.

## Figures and Tables

**Table 1:** Definitions of terms.

Term	Definition	References
Artificial Intelligence (AI)	Computational approach where a computer algorithm automatically develops a model that transforms input data to output without using rules defined by humans.	[3,4,9,10,11,12,13]
Machine learning (ML)	ML is a sub-field of AI. Classical ML methods require input data to have well defined sets of variables in the format of structured data (features).	[2,4,5,6,7,9,13,14,15,16,17,18,19,20,21,22]
Deep learning (DL)	DL is an emerging sub-field of ML where the DL algorithm can take raw data, such as images, as input and “learn” to define its own features needed for computing the outcome.	[2,3,11,23,24,25,26,27]
Ground truth	A number of labelled data sets with known information employed to train machine learning algorithms (e.g., In radiation oncology, manual segmentation by clinicians or trained personnel.	[7,13]
Radiogenomics (in the broader oncology context)	State-of-the-art science in the field of individualised medicine. Radiogenomics combines a large volume of quantitative data extracted from medical images with individual genomic phenotypes and constructs a prediction model through deep learning to stratify patients, guide therapeutic strategies, and evaluate clinical outcomes.	[2,13,14,28]
Radiogenomics (in the radiation oncology context)	Radiogenomics has two goals: (1) develop an assay to predict which cancer patients are most likely to develop radiation injuries resulting from radiotherapy, and (2) obtain information about the molecular pathways responsible for radiation-induced normal tissue toxicities with the ultimate goal of improving oncologic outcomes.	[13,29,30,31,32,33,34]
